# Biological Restoration in Pediatric Dentistry: A Brief Insight

**DOI:** 10.5005/jp-journals-10005-1264

**Published:** 2015-02-09

**Authors:** Indira MD, Kanika Singh Dhull, B Nandlal, Praveen Kumar PS, Rachita Singh Dhull

**Affiliations:** Senior Lecturer, Department of Pedodontics and Preventive Dentistry, JSS Dental College, JSS University, Mysore, Karnataka, India; Reader, Department of Pedodontics and Preventive Dentistry, Kalinga Institute of Dental Sciences, KIIT University, Bhubaneswar Odisha, India; Professor, Department of Pedodontics and Preventive Dentistry, JSS Dental College, JSS University, Mysore, Karnataka, India; Assiatant Professor, Department of Dentistry, Mysore Medical College, Mysore Karnataka, India; Fellow, Department of Pediatric Nephrology, Detroit Medical Centre Michigan, United States

**Keywords:** Biological restoration, Early childhood caries, Primary teeth, Tooth bonding.

## Abstract

Dental caries is the most prevalent disease in humans, especially during early childhood. The restoration of such an extensive carious lesion should be done properly to reestablish their anatomy and hence their masticatory, phonetic, esthetic and space-maintainer functions in the dental arches. The consequences of premature loss of primary teeth are well known, namely the loss of vertical dimension of occlusion, tongue thrusting and mouth breathing habits, which can be the sources of future malocclusion. Satisfactory restoration of these teeth, improving esthetics, along with the management of space and function has always been a challenge for pediatric dentist. An ever increasing demand for esthetics has led to innovation and development of newer treatment modalities for these problems. In an attempt to widen the treatment options as biologically and conservatively as possible, tooth structure is used as a restorative material to rehabilitate severely destroyed tooth crowns. This technique consists of bonding sterile dental fragments, obtained either from the patient or from a tooth bank, to the teeth. Such a technique was termed as ‘biological restoration’.

This article aims at reviewing the evolution, techniques and outcome of such biological restorations.

**How to cite this article:** MD Indira, Dhull KS, Nandlal B, Kumar PSP, Dhull RS. Biological Restoration in Pediatric Dentistry: A Brief Insight. Int J Clin Pediatr Dent 2014;7(3):197-201.

## INTRODUCTION

Dental caries is the most prevalent disease in humans especially during early childhood. Early childhood caries is a major health problem t hat causes significant pain and psychological trauma to young children.^1^ A study on dental caries prevalence among preschool children revealed that caries prevalence is 54.1 and 23% of children had caries in anterior teeth.^2^ Early childhood caries is usually presented with extensive multi-surface involvement of teeth. Restoration of extensively destroyed carious teeth has always been a challenge to pediatric dentist. In the past the only option for severely decayed anterior teeth was to extract the affected teeth and then replace them with the prosthetic substitute until the permanent tooth erupted.

Conventional restorative procedures for severely damaged teeth require metallic restoration for posterior teeth and a combination of metallic and esthetic restoration for anterior teeth. With growing general awareness many children even as young as 3 years are becoming conscious of their appearance.^3^ The loss of esthetically essential anterior teeth may affect the child's confdence and its normal personality development. Also it may cause abnormal habits and speech difficulties.^3^

Satisfactory restoration of these teeth, improving esthetics along with the management of space and function has been a challenge for pediatric dentist. In an attempt to widen the treatment options to rehabilitate severely destroyed tooth, as biologically and conservatively as possible, several authors have suggested the use of tooth structure available from tooth bank as restorative material.^4-6^

The present article is a brief review on ‘biological restorations’ its advantages, disadvantages and clinical techniques. The first paper reporting the use of fragment of extracted teeth as dental restorative material was published in 1964 by Chosak and Eidelman.^7^ Later in 1991 Santos and Bianchii used the technique of bonding sterile tooth dental fragment to teeth with large coronal destruction and termed the technique as ‘biological restoration’.^8^ Tavares in 1992 first described the technique of biological restoration in primary dentition^4^ (Table 1).

**Table 1:** **Table 1:** Summary of biological restoration techniques done by different authors

*Authors*		*Year*		*Technique*		*Results*	
Santos J, Bianchi J^8^		1991		Biological Restoration of severely damaged teeth with resin bonding systems: case reports		Biological restoration has better sealing and provides no microleakage around the restoration. It has better long-term esthetics and offer more treatment options at difficult clinical problems	
Ramires-Romito ACD et al^6^		2000		Biologic restoration of primary anterior teeth		Biologic restoration shows desirable esthetics and good cervical adaptation	
Mandroli PS^12^		2003		Biologic restoration of primary anterior teeth: a case report		Biologic restoration preserves the integrity of patients natural dentition	
Barcelos R et al ^16^		2003		Biological restorations as an alternative treatment for primary posterior teeth		Biologic restoration shows satisfactory retention, esthetics and mastication.	
Sanches K^9^ et al		2007		Biological restorations as a treatment option for primary molars with extensive coronal destruction – a report of two		Biologic restoration is clinically applicable, viable, cost effective restorative procedures for severely damaged primary crowns	
Grewal N, Reeshu S^18^		2008		Biological restorations: as an alternative esthetic treatment for restoration of severely mutilated primary anterior teeth		Biological restorations is a successful, cost effective alternative esthetic treatment for restoration of severely mutilated primary anterior teeth	

### Technique of Biological Restoration

**Fig. 1:**
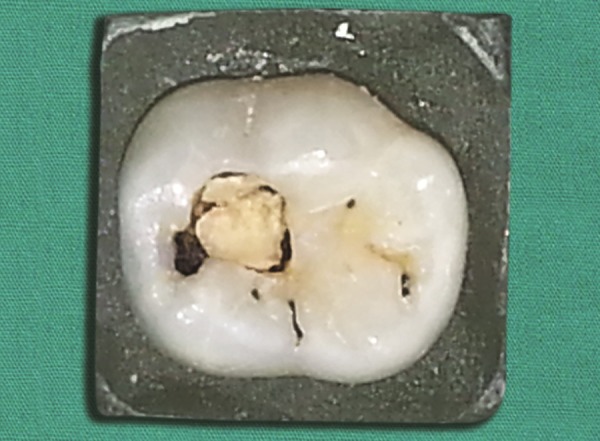
Primary molar with carious lesion

**Fig. 2:**
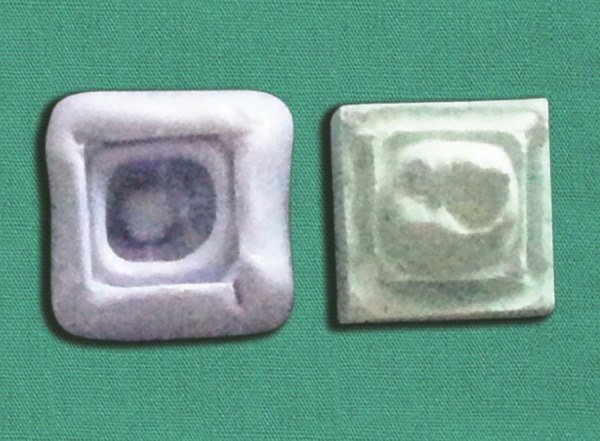
Impression and working model of the prepared cavity

**Fig. 3:**
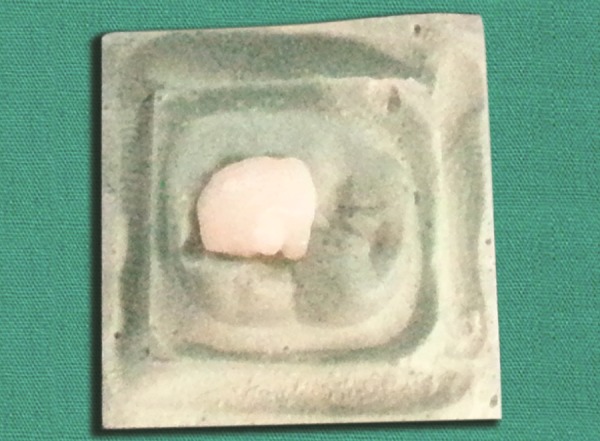
Selected tooth from the tooth bank is adjusted to fit the prepared cavity on the model

**Fig. 4:**
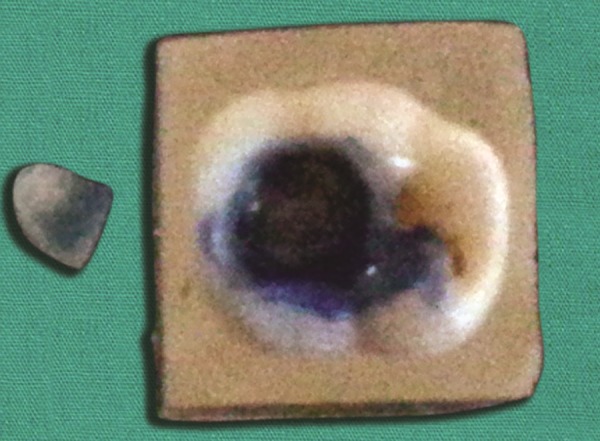
Application of etchant

**Fig. 5:**
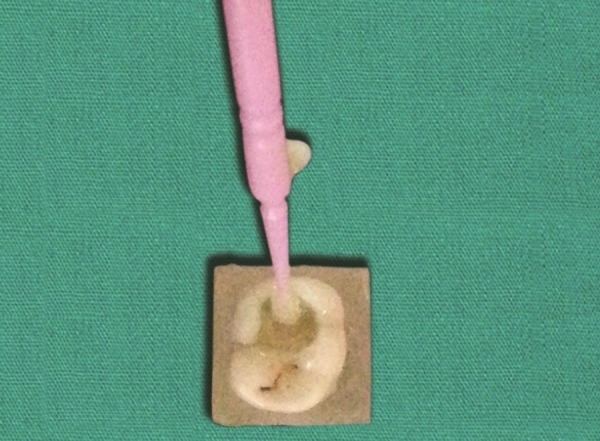
Application of bonding agent

**Fig. 6:**
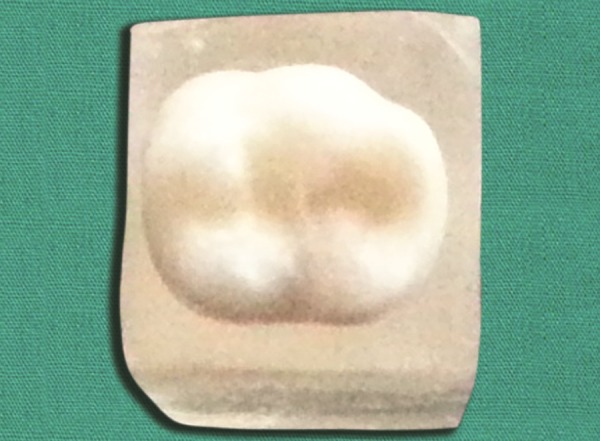
Postoperative fnished restoration

For Carious Posterior Teeth (Figs 1 to 6) as Described by K Sanches et al ^9^

The first step should be to evaluate the extent of carious lesion both clinically and radiographically. This is followed by local anesthesia and rubber dam placement. Remove all the carious lesions and fatten the cavity walls and margins. Protect the tooth with calcium hydroxide liner and glass ionomer cement base; remove rubber dam and make an impression using irreversible hydrocolloid material. On the stone cast obtained measure the mesio-distal, cervico-occlusal and buccolingual dimensions of the tooth using a compass, in order to select an extracted tooth from stock, whose coronal dimensions best fitted the prepared tooth. Color matching is also taken into account. The tooth which is selected, is decoronated and the coronal fragment is adjusted with diamond points at high-speed under air/water spray coolant until it fits the cavity. Interpose articulating paper between the fragment and the cavity in the stone cast to demarcate the areas that need further adjustments. The prepared fragment is autoclaved at 120°C for 20 minutes.

In the second clinical appointment place a rubber dam check the adaptation of the fragment to the tooth. Etch both the cavity and the fragment with a 37% phosphoric acid gel for 30 seconds, rinse and then dried. According to the manufacturer's instructions, bonding agent is applied to the cavity and fragment. Adapt the fragment to the prepared tooth and light cure each surface for 60 seconds. The small imperfections are corrected with light-curing composite resin and the occlusion is checked with articulating paper. Fluoride gel is topically applied to tooth surfaces.

**Fig. 7:**
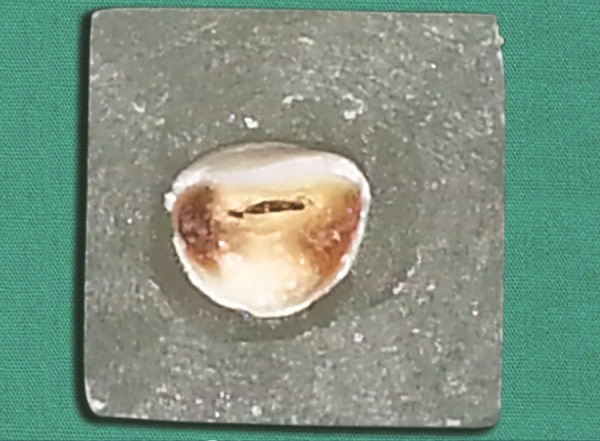
Anterior tooth with extensive caries

**Fig. 8:**
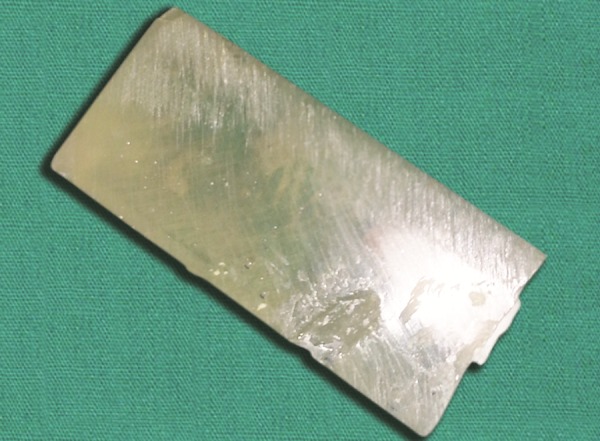
Anterior tooth after endodontic treatment

**Fig. 9:**
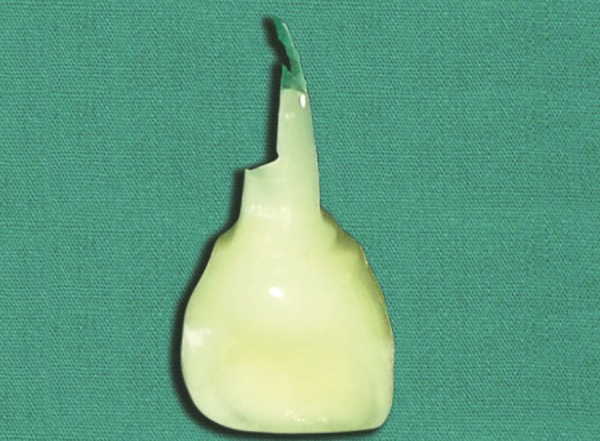
Selection of tooth from tooth bank and modified to fit to the endodontically treated tooth

**Fig. 10:**
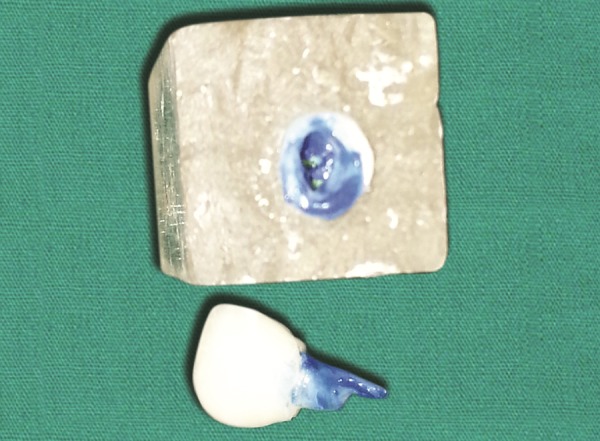
Application of etchant

**Fig. 11:**
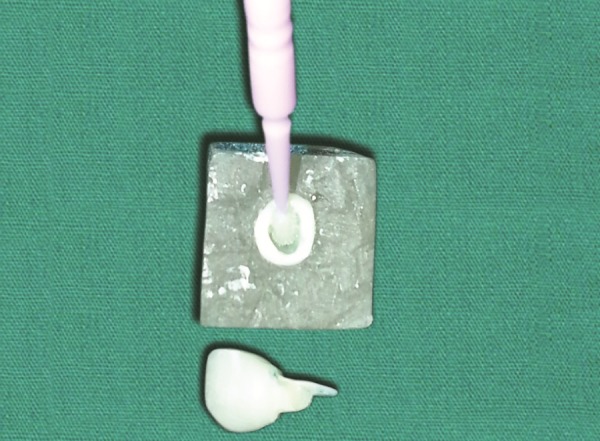
Application of bonding agent

**Fig. 12:**
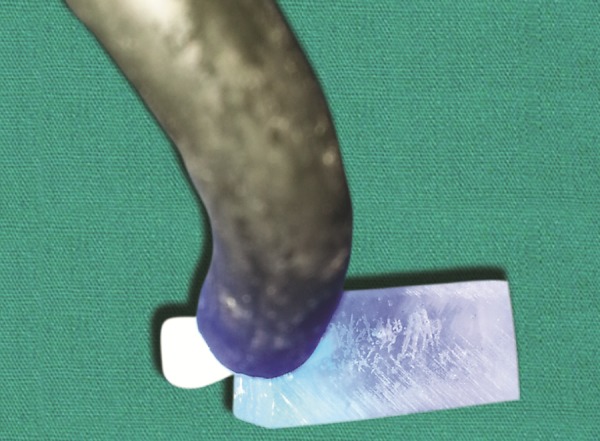
Cementation of the tooth inlay using dual cure resin cement

**Fig. 13:**
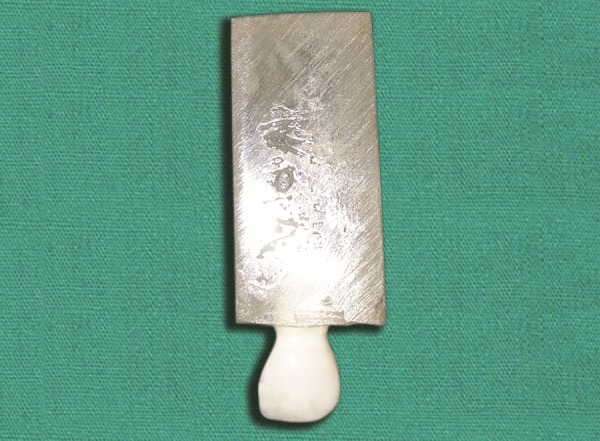
Postoperative fnished restoration

For carious anterior teeth (Figs 7 to 13) as described by Ramires et al.^6^

Accomplish endodontic treatment of all the anterior teeth involved in first appointment. In next session, cleanse and prepare the canals to receive intracanal dentin post. Select the natural post (tooth) and prepare it to fit into the roots. Etch both the root canal and dentin post with 37% phosphoric acid for 15 seconds to receive dentin adhesive. Using dual cure adhesive material cement the dentin post to the root canal. A nonretentive preparation is made ending in chamfer shoulder type margin with rounded corners. Cemented post is protected with the provisional material till next session.

A silicon impression of the prepared teeth is taken to aid in selection of natural crowns in the tooth bank. After autoclave sterilization the prepared crown is cemented with dual cure resin composite. The cervical margins of the restoration is polished with rotary instruments and resin composite polishing disks.

### Indications

- Extensive carious lesion;
  – Insufficient tooth surface to retain amalgam/composite restoration
  – Children with rampant caries
- Following pulpal therapy as an alternative treatment to stainless steel crown/composite resin.

### Advantages

As reported by different authors are as follows:

- The technique is simple, allows the preservation of sound tooth structure and provides excellent esthetics compared to composite resins and stainless steel crowns, especially regarding translucency^9^
- Allows the maintenance of pulpal vitality^10^
- Has a low cost^11^
- Using tooth fragments as restorative material offers superficial smoothness, cervical adaptation and physiologic wear compatible with those of surrounding teeth.^12-14^
- Biological restorations not only mimic the missing part of the oral structures, but are also biofunctional^3^
- Clinical chairtime for fragment bonding procedures is relatively short, which is very interesting when treating pediatric patients^3,6,15,16^
- Restoration is less subjected to extrinsic pigmentation and plaque accumulation when compared to composite resin.^6^

### Disadvantages

As reported by different authors are as follows:

- Though it requires a short clinical chair side time as any indirect restorations, biological restorations require a laboratorial phase that may become a critical step if not properly handled^9^
- Inspite of being simple, the technique requires professional expertise to adequately prepare and adapt the natural crowns to the cavity^9^
- Difficulty in obtaining teeth with the required coronal dimensions^9^
- Difficulty in matching fragment color with tooth remnant color^9^
- Also, having fragments from other people's teeth in their mouth is not a pleasant idea for some patients and many of them refuse to receive this treatment^15^
- Technique is considered difficult for UG students^6^
- The use of very thin fragments where all the dentin is removed lowers the fracture resistance of bonded fragment^6^
- Availability of tooth from tooth bank.^9^ (K Sanches et al 2007).

However, all these factors are not contraindications of the technique.

### Sterilization of Teeth

The best method for sterilization of extracted teeth has not been defined.^16^ Humid steam vapor is the most frequently used technique in biological restoration^6,15^ and most recommended.^17^ It has been verified by means of microbiological culturing and SEM that Humid steam vapor is safe method of eliminating microorganism without interfering with fragment bonding.^16^

Other forms of sterilization of extracted teeth are: ethylene oxide and gamma radiation.

### Factors to be Considered for Biological Restoration^4^

- Time spent on the dental chair
- Total cost of the treatment
- Possibility for the need for repair
- Acceptability by the patient and parents.

## CONCLUSION

There exist no standardized procedures to restore broken down primary anterior teeth to the gingival level. These grossly broken down teeth require a different management solution.

The use of biological restorations over a short composite post provides one of the treatment means. Authors have suggested that not only the children but also the parents are satisfied with the outcome of these restorations.
